# Telomere Length Associations With Clinical Diagnosis, Age, and Polygenic Risk Scores for Anxiety Disorder, Depression, and Bipolar Disorder

**DOI:** 10.1016/j.bpsgos.2022.08.008

**Published:** 2022-09-06

**Authors:** Julian Mutz, Cathryn M. Lewis

**Affiliations:** a Social, Genetic and Developmental Psychiatry Centre, Institute of Psychiatry, Psychology & Neuroscience, King’s College London, London, United Kingdom; b Department of Medical and Molecular Genetics, Faculty of Life Sciences & Medicine, King’s College London, London, United Kingdom

**Keywords:** Aging, Genetics, Mental disorders, Telomeres, UK Biobank

## Abstract

**Background:**

Accelerated biological aging might contribute to the lower life expectancy of individuals with mental disorders. The aim of this study was to characterize telomere length, a biological hallmark of aging, in individuals with mental disorders.

**Methods:**

The UK Biobank is a multicenter community-based observational study that recruited >500,000 middle-aged and older adults. Average leukocyte telomere length (telomere repeat copy number/single-copy gene ratio) was measured using quantitative polymerase chain reaction. Polygenic risk scores (PRSs) were calculated for individuals of European ancestry. We estimated differences in telomere length between individuals with anxiety disorder, depression, or bipolar disorder and people without mental disorders and examined associations with psychotropic medication use, age, and PRSs for these 3 disorders.

**Results:**

The analyses included up to 308,725 participants. Individuals with depression had shorter telomeres than people without mental disorders (β = −0.011, 95% CI, −0.019 to −0.004, Bonferroni-corrected *p* = .027). Associations between bipolar disorder and telomere length differed by lithium use. There was limited evidence that individuals with an anxiety disorder had shorter telomeres. There was no evidence that associations between age and telomere length differed between individuals with and without these disorders. PRSs for depression, but not anxiety disorder or bipolar disorder, were associated with shorter telomeres (β = −0.006, 95% CI, −0.010 to −0.003, Bonferroni-corrected *p* = .001).

**Conclusions:**

Differences in telomere length were observed primarily for individuals with depression or bipolar disorder and in individuals with a higher PRS for depression. There was no evidence that the association between age and telomere length differed between individuals with and without an anxiety disorder, depression, or bipolar disorder.

Telomeres are repetitive nucleoprotein complexes at the chromosome ends that play an important role in maintaining genomic stability. Telomeres shorten with each cell division and therefore represent a biological marker of replicative history and cellular age (1,2). Although telomere length is highly heritable (3), age-related attrition results from biological and environmental factors, including lifestyle and chronic stress (4). Telomere attrition has been associated with an increased risk of age-related diseases. Mendelian randomization analyses in the UK Biobank, a major biomedical database, suggested that telomere length had a widespread influence on biomedical traits, disease risk, multiple body systems, and life expectancy (5).

Individuals with mental disorders have an increased prevalence of age-related diseases and a lower life expectancy (6). They also show signs of accelerated biological aging, including advanced brain aging (7), changes in DNA methylation (8), greater levels of inflammation (9), elevated frailty (10), and differences in physiological markers such as grip strength (11, 12, 13). Telomere length as a molecular marker of cellular age could provide insight into the relation between mental health and accelerated biological aging. Data from a meta-analysis suggested that individuals with anxiety disorders, depressive disorders, and posttraumatic stress disorder had shorter telomeres than people without these disorders (14). Findings regarding bipolar disorder have been inconsistent (15), with some studies observing longer telomeres in patients (14), likely due to lithium treatment (16). Patients with bipolar disorder not exposed to lithium had shorter telomeres than patients who had been treated with lithium (17). Most previous studies have had limited sample sizes, and few studies have included cross-disorder comparisons within the same database.

There has also been little exploration of associations between telomere length and genetic risk for mental disorders. Although multiple studies have examined polygenic scores for telomere length to predict mental disorders (18), there has been limited research on polygenic risk scores (PRSs) for mental disorders to predict telomere length. Preliminary studies found that unaffected first-degree relatives of individuals with bipolar disorder had shorter telomeres than healthy control subjects (19,20). Similarly, a small cross-sectional study found that daughters of mothers with depression had shorter telomeres than daughters of never-depressed mothers (21). Although these findings suggest that an increased genetic risk for mental disorders may affect telomere length, these studies were limited by modest sample sizes and cannot fully disentangle genetic and environmental risk factors. A depression PRS was not associated with telomere length or telomere attrition rate in 2032 adults ages 18 to 65 years (18). Finally, a study of 290 adults without depression also found no evidence that PRSs for depression, bipolar disorder, or schizophrenia were associated with telomere length (22).

The UK Biobank provides an unprecedented resource to investigate health and aging, with the world’s largest database of leukocyte telomere length measurements. The aim of this study was to examine cross-sectional differences in telomere length between individuals with a history of anxiety disorder, depression, or bipolar disorder and people without mental disorders and to examine associations between telomere length, psychotropic medication use, age, and PRSs for these disorders.

## Methods and Materials

### Study Population

The UK Biobank is a prospective study of >500,000 UK residents ages 37 to 73 years at baseline who were recruited between 2006 and 2010. The study rationale and design have been described elsewhere (23). Briefly, individuals registered with the UK National Health Service and living within a 25-mile (∼40 km) radius of one of the 22 assessment centers were invited to participate. Participants provided data on their sociodemographic characteristics, health behaviors, and medical history; underwent physical examination; and had blood and urine samples taken. Linked hospital inpatient records are available for most participants, and primary care data are available for half of the participants. A third of participants completed an online follow-up Mental Health Questionnaire between 2016 and 2017.

### Leukocyte Telomere Length

Details of the measurement of leukocyte telomere length (UK Biobank data fields 22191 and 22192), including extensive quality control and technical adjustments, have been reported elsewhere (24). Briefly, relative telomere length was measured using a validated quantitative polymerase chain reaction assay that expresses telomere length as the ratio of the telomere repeat copy number (T) relative to a single-copy gene (S) that encodes hemoglobin subunit beta. The T/S ratio is proportional to an individual’s average telomere length (25). The amounts of T and S were measured within each reaction and were calculated relative to a calibrator sample of pooled DNA from 20 individuals that was included in every run. Each measurement run included 47 samples in duplicate, a no-template control, and the calibrator sample in quadruplicate (24). Measurements were adjusted for operational and technical parameters (polymerase chain reaction machine, staff member, enzyme batch, primer batch, temperature, humidity, primer batch × polymerase chain reaction machine, primer batch × staff member, A260/A280 ratio of the DNA sample, and A260/A280 ratio squared), log_e_ transformed (due to non-normality), and Z-standardized (to allow direct comparisons with other studies). For descriptive purposes, T/S ratio was converted to base pairs using the following formula: base pairs = 3274 + 2413 × ([T/S − 0.0545]/1.16) (26).

### Mental Disorders

We identified individuals with lifetime anxiety disorder, depression, or bipolar disorder using our previously reported criteria (11, 12, 13). Data sources included the modified Composite International Diagnostic Interview Short Form, self-report questions on (hypo)mania and a question on psychiatric diagnoses (field 20544) that were assessed as part of the Mental Health Questionnaire, the nurse-led baseline interview in which participants reported medical diagnoses (field 20002), hospital inpatient records (ICD-10 codes), primary care records (Read Version 2 or Clinical Terms Version 3 codes), and self-report questions on mood disorders from the baseline assessment (field 20126). Participants were included in the group of individuals with a mental disorder if at least one of the data sources indicated a history of mental disorder. Individuals with psychosis were excluded from all groups, and individuals with bipolar disorder were excluded from the anxiety disorder group due to their increased risk of physical multimorbidity (27,28). The depression and bipolar disorder groups were mutually exclusive. Individuals could be included in both the anxiety disorder and the depression groups.

Individuals in the nonpsychiatric comparison group had no mental disorders: 1) did not report schizophrenia, depression, mania/bipolar disorder/manic depression, anxiety/panic attacks, obsessive-compulsive disorder, anorexia/bulimia/other eating disorder, or posttraumatic stress disorder at the baseline interview; 2) reported no psychiatric diagnoses on the Mental Health Questionnaire; 3) did not report current psychotropic medication use at baseline (field 20003) (24); 4) had no ICD-10 Chapter V code in their hospital inpatient records (F20-F99), except for organic causes or substance use; 5) had no diagnostic codes for mental disorders in their primary care records (25); 6) were not classified as individuals with probable mood disorder at the baseline assessment; 7) had no Patient Health Questionnaire-9 or Generalised Anxiety Disorder Assessment sum score of ≥5; 8) never felt worried, tense, or anxious for most of a month or longer (field 20421); and 9) were not identified as individuals with a history of mental disorder based on the Composite International Diagnostic Interview Short Form and questions on (hypo)manic symptoms (12,13).

### Genetic Quality Control

Genetic quality control was performed as described previously (29). Individuals were excluded as recommended by the UK Biobank due to unusual levels of missingness (>5%) or heterozygosity (23). Using the genotyped single nucleotide polymorphisms, individuals with call rate of <98%, who were genetically related to another individual in the dataset (KING *r* < 0.044, equivalent to removing third-degree relatives and closer) (30) or whose self-reported and genotypic sex did not match (X chromosome homozygosity [*F*_X_] < 0.9 for phenotypic males, *F*_X_ > 0.5 for phenotypic females) were also excluded. To account for familial correlation, removal of relatives was performed using a “greedy” algorithm, which minimizes exclusions (e.g., by excluding the child in a mother-father-child trio) (31). All analyses were limited to individuals of European ancestry, as defined by 4-means clustering on the first 2 genetic principal components (PCs) provided by the UK Biobank (32). PC analysis was also performed on the European-only subset of the data using FlashPCA2 (33).

### Polygenic Risk Scores

PRSs for anxiety disorder, depression, and bipolar disorder were calculated using PRSice version 2 (34). This method involves calculating PRSs as the sum of risk alleles weighted by single nucleotide polymorphism effect sizes from independent genome-wide association study summary statistics (Table S1). Clumping was performed to remove single nucleotide polymorphisms in high linkage disequilibrium (defined as *r*^2^ ≥ 0.1 within 250 kb on each side), as linkage disequilibrium can falsely inflate polygenic scores. PRSs were calculated at 11 *p*-value thresholds (5 × 10^−8^, 1 × 10^−5^, 1 × 10^−3^, .01, .05, .1, .2, .3, .4, .5, and 1), and the PRSs’ most predictive threshold was selected for regression analyses. All individual-level PRSs were standardized prior to analyses.

### Covariates

Covariates were identified from previous research and included age (24), sex (24), white blood cell count (24), Townsend deprivation index (35), physical activity (36), smoking status (37), body mass index (38), body fat percentage (39), and C-reactive protein (40). Details of these data fields are presented in Table S2. For the analyses of PRSs, covariates included the first 6 ancestry-informative population PCs, batch number, and assessment center.

### Statistical Analyses

Regression analyses were performed in R (version 3.6.2).

Sample characteristics were summarized using means and standard deviations or counts and percentages. Differences in T/S ratio (log z adjusted) between individuals with anxiety disorder, depression, or bipolar disorder and the comparison group without mental disorders were estimated using ordinary least squares regression (±95% confidence intervals). For these analyses, we fitted minimally adjusted models that included age and sex and fully adjusted models that included all covariates. Age-related differences in T/S ratio (log z adjusted) were estimated using generalized additive models within the “mgcv” package (41) in R. Finally, associations between T/S ratio (log z adjusted) and PRSs for anxiety, depression, and bipolar disorder were estimated using ordinary least squares regression. These models included 6 PCs, the batch number, and the assessment center.

We calculated adjusted *p* values to correct for multiple testing. Two methods were used: 1) Bonferroni (Bonf) and 2) Benjamini-Hochberg (BH) (42), all two-tailed, with α = 0.05 and a false discovery rate of 5%, respectively. *p* Values were corrected for 3 to 12 tests. We have opted for this approach because the Bonferroni correction may be too conservative and may potentially lead to a high number of false negatives.

### Additional Analyses

We repeated our main analyses 1) with the bipolar disorder group stratified by current lithium use, 2) comparing all individuals with mental disorders stratified by lithium use to individuals without mental disorders, and 3) stratified by antidepressant and antipsychotic medication use. For medication codes, see our previous studies (11, 12, 13). As a sensitivity analysis, we excluded individuals with comorbid depression and anxiety disorder. Finally, we stratified the PRS analyses by case status to assess the association between PRSs independent of diagnosis and treatment-related confounders.

## Results

After quality control exclusions and restricting our sample to individuals of European ancestry, 458,078 participants (of 502,476) had data on both telomere length and PRSs. We retained up to 308,725 participants with complete data on all covariates. A total of 41,524 individuals had a lifetime anxiety disorder, 84,965 had lifetime depression, and 3449 had bipolar disorder. The sample characteristics of each group are shown in Table 1. Compared with individuals without mental disorders, individuals with anxiety disorder, depression, or bipolar disorder were younger, more likely female, lived in more deprived neighborhoods, engaged in less physical activity, were more likely to smoke, had an elevated body mass index and body fat percentage, were more likely obese, had an elevated white blood cell count, and had higher C-reactive protein levels.

**Table 1:** Sample Characteristics of Individuals With and Without Mental Disorders

Characteristics	Anxiety Disorder, *n* = 41,524	Depression, *n* = 84,965	Bipolar Disorder, *n* = 3449	No Disorder, *n* = 223,760
T/S Ratio, Log Z Adjusted	0.02 (0.99)	0.02 (0.99)	0.01 (1.01)	−0.01 (1.00)
Telomere Length, Base Pairs	4897.74 (271.66)	4896.79 (270.31)	4895.22 (270.45)	4888.25 (271.99)
Age, Years	56.04 (7.86)	55.55 (7.88)	54.76 (7.97)	56.77 (8.08)
Sex				
Female	27,316 (65.8%)	55,322 (65.1%)	1915 (55.5%)	111,064 (49.6%)
Male	14,208 (34.2%)	29,643 (34.9%)	1534 (44.5%)	112,696 (50.4%)
Neighborhood Deprivation	−1.36 (3.04)	−1.22 (3.06)	−0.71 (3.21)	−1.68 (2.87)
Walking^a^	5.31 (1.98)	5.32 (1.99)	5.37 (2.05)	5.43 (1.91)
Moderate Activity^a^	3.53 (2.35)	3.52 (2.36)	3.64 (2.42)	3.66 (2.31)
Vigorous Activity^a^	1.74 (1.91)	1.74 (1.92)	1.87 (2.03)	1.92 (1.96)
Smoking Status				
Never	21,393 (51.5%)	42,648 (50.2%)	1548 (44.9%)	125,978 (56.3%)
Former	15,494 (37.3%)	31,614 (37.2%)	1248 (36.2%)	77,482 (34.6%)
Current	4637 (11.2%)	10,703 (12.6%)	653 (18.9%)	20,300 (9.1%)
BMI	27.27 (4.99)	27.63 (5.11)	28.02 (5.33)	27.14 (4.45)
Body Fat Percentage	32.68 (8.54)	32.94 (8.61)	31.90 (8.80)	30.45 (8.34)
Obesity				
Underweight, BMI < 18.5	281 (0.7%)	442 (0.5%)	17 (0.5%)	963 (0.4%)
Normal, 18.5 ≤ BMI < 25	14,593 (35.1%)	27,774 (32.7%)	1052 (30.5%)	75,326 (33.7%)
Overweight, 25 ≤ BMI < 30	16,684 (40.2%)	34,299 (40.4%)	1346 (39.0%)	98,576 (44.1%)
Obese, 30 ≤ BMI < 35	6959 (16.8%)	15,335 (18.0%)	709 (20.6%)	36,773 (16.4%)
Severely Obese, BMI ≥ 35	3007 (7.2%)	7115 (8.4%)	325 (9.4%)	12,122 (5.4%)
White Blood Cell Count^b^	6.90 (1.92)	6.96 (1.96)	7.18 (2.47)	6.81 (2.03)
C-Reactive Protein^b^	2.60 (4.26)	2.69 (4.31)	2.80 (4.12)	2.41 (4.14)
Antidepressant Use				
No	32,645 (78.6%)	66,508 (78.3%)	2408 (69.8%)	223,760 (100.0%)
Yes	8879 (21.4%)	18,457 (21.7%)	1041 (30.2%)	0 (0.0%)
Antipsychotic Use				
No	41,293 (99.4%)	84,592 (99.6%)	3204 (92.9%)	223,760 (100.0%)
Yes	231 (0.6%)	373 (0.4%)	245 (7.1%)	0 (0.0%)
Lithium Use				
No	41,483 (99.9%)	84,849 (99.9%)	3144 (91.2%)	223,760 (100.0%)
Yes	41 (0.1%)	116 (0.1%)	305 (8.8%)	0 (0.0%)

Data are presented as *n* (%) or mean (SD).

BMI, body mass index.

a Number of days per week engaging in these activities for 10+ minutes continuously.

b Units: white blood cell count, × 10^9^ cells/L; C-reactive protein, mg/L.

**Figure 1:**
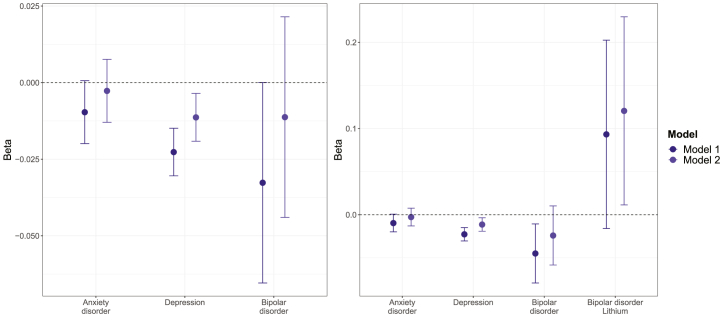
Average telomere repeat copy number/single-copy gene ratio (log z adjusted) in individuals with mental disorders compared with individuals without mental disorders (reference group). Estimates shown are ordinary least squares regression beta coefficients and 95% confidence intervals. Model 1—adjusted for age and sex; model 2—adjusted for age, sex, white blood cell count, Townsend deprivation index, physical activity, smoking status, body mass index, body fat percentage, and C-reactive protein.

Average telomere length (T/S ratio log z adjusted) in individuals with and without mental disorders is shown in Figure S1. After adjusting for age and sex (model 1) and other potential confounders (model 2), we observed that individuals with mental disorders had slightly shorter telomeres (Figure 1). However, this difference was only statistically significant for the comparison between individuals with depression and individuals without mental disorders (fully adjusted β = −0.011, 95% CI, −0.019 to −0.004, *p*_Bonf_ = .027) (Table 2).

**Table 2:** Telomere Repeat Copy Number/Single-Copy Gene Ratio (log z adjusted) in Individuals With Mental Disorders

Term	Model 1				Model 2			
	β	95% CI	*p*_Bonf_	*p*_BH_	β	95% CI	*p*_Bonf_	*p*_BH_
No Disorder	Ref				Ref			
Anxiety Disorder	−0.010	−0.020 to 0.001	.396	.099	−0.003	−0.013 to 0.008	>.999	.609
Depression	−0.023	−0.030 to −0.015	<.001	<.001	−0.011	−0.019 to −0.004	.027	.014
Bipolar Disorder	−0.033	−0.065 to 0.000	.303	.099	−0.011	−0.044 to 0.022	>.999	.601
No lithium	−0.045	−0.079 to −0.011	.082	.027	−0.024	−0.058 to 0.010	>.999	.193
Lithium	0.093	−0.016 to 0.203	.754	.126	0.121	0.011 to 0.230	.243	.061

Model 1 adjusted for age and sex; model 2 adjusted for age, sex, white blood cell count, Townsend deprivation index, physical activity, smoking status, body mass index, body fat percentage, and C-reactive protein. *p* Values corrected for 6 (main analysis) and 8 (bipolar disorder cases stratified by lithium use) tests.

β, ordinary least squares regression beta coefficient; BH, Benjamini-Hochberg; Bonf, Bonferroni; Ref, reference group.

When stratifying individuals with bipolar disorder by current lithium use, we found that after adjusting for age and sex, telomeres were shorter in individuals who did not use lithium (adjusted β = −0.045, 95% CI, −0.079 to −0.011, *p*_BH_ = .027) than in individuals without mental disorders. Individuals with bipolar disorder who used lithium had slightly longer telomeres than individuals without mental disorders (fully adjusted β = 0.121, 95% CI, 0.011– 0.230, *p*_BH_ = .061), although this difference was not statistically significant after multiple testing corrections. Comparing all individuals with mental disorders (i.e., anxiety disorder, depression, or bipolar disorder) stratified by lithium use to individuals without mental disorders, we found that individuals who did not use lithium had shorter telomeres than the comparison group (fully adjusted β = −0.009, 95% CI, −0.016 to −0.002, *p*_BH_ = .035). There was no evidence of statistically significant differences in telomere length between individuals with mental disorders who used lithium and individuals without mental disorders (fully adjusted β = 0.060, 95% CI, −0.033 to 0.152, *p*_BH_ = .277) (Figure S2 and Table S3). Individuals with anxiety disorder or depression who reported antidepressant medication use had shorter telomeres than individuals without mental disorders (Figure S3 and Table S4). Finally, individuals with depression who used antipsychotic medications had shorter telomeres than both individuals without mental disorders and individuals with depression who did not report antipsychotic medication use (Figure S4 and Table S5).

**Figure 2:**
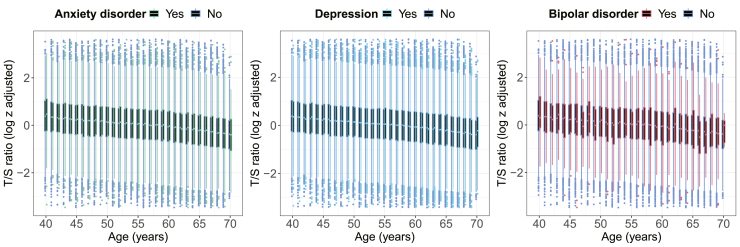
Age-related differences in average telomere repeat copy number/single-copy gene (T/S) ratio (log z adjusted) in individuals with and without mental disorders. T/S ratio values below the 0.01st or above the 99.99th percentile not shown.

As expected, telomere length reduced with age (Figure 2). These data presented for 5-year age groups are shown in Figure S5. There was no evidence that age-related differences in telomere length differed between individuals with anxiety disorder, depression, or bipolar disorder and people without mental disorders (*p* values between .39 and .94) (Figure S6).

**Figure 3:**
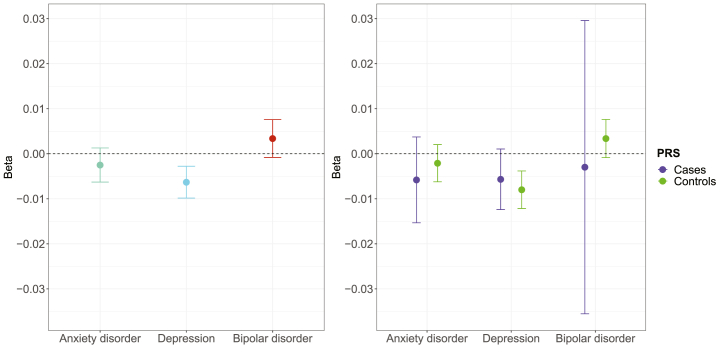
Associations between average telomere repeat copy number/single-copy gene ratio (log z adjusted) and polygenic risk scores (PRSs) for anxiety disorder, depression, and bipolar disorder. All analyses were adjusted for the first 6 ancestry-informative population principal components, the batch number, and the assessment center.

The distribution of PRSs in individuals with and without mental disorders is shown in Figure S7, confirming that there were small to moderate increases in PRSs in individuals with mental disorders. There was little evidence of an association between the PRSs for anxiety disorder, depression, or bipolar disorder and telomere length (Figure S8). In a regression model, the PRS for depression was associated with shorter telomeres (adjusted β = −0.006, 95% CI, −0.010 to −0.003, *p*_Bonf_ = .001). There was no evidence that the PRSs for anxiety disorder (adjusted β = −0.002, 95% CI, −0.006 to 0.001, *p*_Bonf_ = .589) or bipolar disorder (adjusted β = 0.003, 95% CI, −0.001 to 0.008, *p*_Bonf_ = .342) were associated with telomere length (Figure 3). When stratifying these analyses by case status, the PRS for depression was only statistically significantly associated with shorter telomeres in individuals without mental disorders (adjusted β = −0.008, 95% CI, −0.012 to −0.004, *p*_Bonf_ = .001) (Table S6).

### Sensitivity Analysis

A total of 53,780 individuals had a history of depression without comorbid anxiety disorder, while 14,829 individuals had an anxiety disorder without comorbid depression. Individuals with depression had shorter telomeres (fully adjusted β = −0.015, 95% CI, −0.025 to −0.006, *p*_Bonf_ = .004) (Figure S9), and this difference was slightly greater than in the main analysis. There was no evidence of a difference in telomere length between individuals with anxiety disorder and individuals without mental disorders (fully adjusted β = 0.004, 95% CI, −0.012 to 0.021, *p*_Bonf_ > .999) (Table S7).

Finally, individuals with depression who reported antidepressant medication use had shorter telomeres than individuals with depression who did not report medication use relative to the comparison group without mental disorders (Figure S10 and Table S8). There was no evidence of an association between telomere length and anxiety disorder, irrespective of antidepressant medication use.

## Discussion

Individuals with a lifetime history of depression had slightly shorter telomeres than people without mental disorders. There was only limited evidence that telomere length differed between individuals with an anxiety disorder or bipolar disorder and people without mental disorders. Notably, there was some evidence that lithium use was associated with elongated telomeres in individuals with bipolar disorder, while individuals with bipolar disorder who did not use lithium had shorter telomeres. Antidepressant and antipsychotic medication use was associated with reduced telomere length in individuals with depression. Age-related differences in telomere length did not differ between individuals with and without mental disorders. PRSs for depression were associated with shorter telomeres. There was no evidence that PRSs for anxiety disorder or bipolar disorder were associated with telomere length.

The observation that depression was associated with shorter telomeres in the UK Biobank is consistent with data from meta-analyses (14,43,44). Although meta-analyses also provided evidence of an association between anxiety disorders and shorter telomeres (14,45), we did not observe a statistically significant difference between individuals with an anxiety disorder and people without mental disorders. This discrepancy could be due to differences in the definition of anxiety disorder, including the specific diagnoses being considered, severity and chronicity, or depression comorbidity. Data from 2 meta-analyses found no association between bipolar disorder and telomere length (14,46). However, the most recent meta-analysis suggested that patients with bipolar disorder had shorter telomeres than participants in the control group (47). Inconsistencies between studies could relate to differences in sample characteristics. For example, a recent study found that patients with bipolar disorder type I, but not bipolar disorder type II, had shorter telomeres than healthy control subjects (48). Another study did not observe group differences in telomere length between bipolar disorder subtypes but was likely underpowered (*n* = 119 vs. *n* = 12, respectively) (17).

Our finding that lithium use modified the direction of association between bipolar disorder and telomere length is consistent with previous observations that lithium treatment was associated with increased telomere length (19,49) and that telomere length positively correlated with duration of lithium treatment (16,49). A recent study found that patients with bipolar disorder who had never been treated with lithium had shorter telomeres than healthy control subjects, while patients treated with lithium had longer telomeres than the never-treated patients, although not longer than those of healthy control subjects (17). Our finding that psychotropic medication use was associated with reduced telomere length in individuals with depression aligns with a preliminary study (*n* = 40) suggesting that antidepressant use was associated with shorter telomeres, independent of depression diagnosis and current depression severity (22). However, caution is warranted in interpreting this finding because we did not consider other patient and treatment-related characteristics, such as depression severity, that correlate with medication use. Data from a Dutch cohort found that the duration and severity of depression, but not antidepressant medication use, were associated with shorter telomeres in individuals with a history of depression (26). Future studies in the UK Biobank could explore to what extent other patient-, illness-, and treatment-related factors, including length of illness, number of episodes, history of suicide attempt, duration of treatment, and number of previous hospitalizations, explain differences in telomere length.

Although previous research suggested that age-related decline in telomere length was greater in individuals with chronic stress or comorbidities (50), we observed a similar association between telomere length and age in individuals with and without mental disorders.

Previous research suggested that depression PRSs were not associated with telomere length or telomere attrition rate in 2032 adults of ages 18 to 65 years (18). A study of 290 adults without depression also found no evidence that PRSs for depression, bipolar disorder, or schizophrenia were associated with telomere length (22). We found that PRSs for depression, but not for anxiety disorder or bipolar disorder, were associated with shorter telomeres, although the strength of this association was negligible. Our finding that depression PRSs were associated with telomere length only in individuals without mental disorders could be explained by the lower sample size in the case group and warrants replication.

Several mechanisms could explain telomere length differences between individuals with depression or bipolar disorder and people without mental disorders. Individuals with these disorders engage in less healthy lifestyle behaviors that are known to affect telomere length, for example, physical inactivity (36) and smoking (37). Shorter telomeres could also be due to biological mechanisms, including overactivation of the hypothalamic-pituitary-adrenal axis and autonomic nervous system, increased levels of inflammation and oxidative stress, or poor metabolic health in depression and bipolar disorder (51). Finally, increased rates of physical comorbidities in individuals with mental disorders (52) may also contribute to reduced telomere length. There is conflicting evidence regarding potential mechanisms linking antidepressant medications and telomere length. A potential mechanism linking antidepressant medication use with shorter telomeres is increased blood cell proliferation (53), which may result in telomere shortening. However, it is also possible that antidepressant medication use increases telomerase activity, the enzyme that maintains and elongates telomeres (54).

In contrast to most previous studies, we examined associations between telomere length and mental disorders in the same data using a shared comparison group of individuals without mental disorders, allowing for cross-disorder comparison. Our study also included a considerably larger number of individuals with mental disorders. Indeed, the UK Biobank is by far the largest data resource with measured telomere length, which allowed us to adjust for a range of potential confounders.

Our observational study has limitations. The age range was limited to middle-aged and older adults (most between 40 and 69 years of age). A previous Dutch study found no difference in telomere length between individuals with current depression who were 60 years or older compared to never-depressed individuals, suggesting that our findings might not extrapolate to late-life depression (55). Similarly, the exclusion of younger participants could have contributed to certain negative findings. For example, a recent study found shorter telomere length only in younger individuals with euthymic bipolar disorder (56). For the cross-sectional analyses, our ability to draw causal conclusions was limited. Longitudinal studies have found that major depressive disorder (57) or persistent internalizing disorders in men (58) predicted reduced telomere length, although not all studies found evidence of a prospective association between mental disorders and telomere length (44,59). A large study of 67,306 individuals of ages 20–100 years from the Danish general population found no evidence that telomere length predicted depression prospectively or that genetically shorter telomeres predicted depression. Nevertheless, the authors observed that depression was associated with shorter telomeres cross-sectionally, which could be explained by depression causing shorter telomeres or residual confounding (60). Telomeres were measured from leukocyte DNA, and findings might differ when examining other tissues. However, research suggests that leukocyte telomeres correlate well with telomere length measured in other tissues (61). Absolute telomere length, which could have led to lower interexperiment variability, was not directly measured in this study. Finally, only data on average telomere length were available; therefore, we could not examine whether individuals with and without mental disorders differed in their shortest telomeres, which determine telomere dysfunction and limit cell proliferation (25).

### Conclusions

Cross-sectional differences in telomere length were observed primarily for individuals with depression or bipolar disorder and in individuals with a higher PRS for depression. Psychotropic medication use modified associations between mental disorders and telomere length, though further research is needed to dissect the potential effects of medication use and correlated patient and illness-related factors. There was little evidence that the association between age and leukocyte telomere length differed between middle-aged and older adults with a lifetime history of anxiety disorder, depression, or bipolar disorder and individuals without mental disorders.

## Supplementary Material

Supplemen

Key Resources Table
