# Supplementary material for: Telomere Length Associations With Clinical Diagnosis, Age, and Polygenic Risk Scores for Anxiety Disorder, Depression, and Bipolar Disorder
Source: Biol Psychiatry Glob Open Sci. 2022 Sep 6;3(4):1012–20. doi: 10.1016/j.bpsgos.2022.08.008 (PMC10593885; doi:10.1016/j.bpsgos.2022.08.008)
Supplement: Supplemen [file mmc1.pdf]

SUPPLEMENTARY INFORMATION

Telomere Length Associations With Clinical Diagnosis, Age and Polygenic Risk Scores for Anxiety Disorder, Depression and Bipolar Disorder

Mutz and Lewis

Table of contents:

GWAS summary statistics ... 2
Covariates ... 3
T/S ratio in individuals with mental disorders ... 4
T/S ratio in individuals with any mental disorder by lithium use ... 5
  Figure: T/S ratio by lithium use ... 5
  Table: T/S ratio by lithium use ... 6
T/S ratio in individuals with mental disorders by antidepressant medication use ... 7
  Figure: T/S ratio by antidepressant medication use ... 7
  Table: T/S ratio by antidepressant medication use ... 8
T/S ratio in individuals with mental disorders by antipsychotic medication use ... 9
  Figure: T/S ratio by antipsychotic medication use ... 9
  Table: T/S ratio by antipsychotic medication use ... 10
Associations between age and T/S ratio in individuals with and without mental disorders ... 11
  5-year age groups ... 11
  Generalised additive models ... 12
Polygenic risk scores in individuals with mental disorders ... 13
Associations between T/S ratio and polygenic risk scores ... 14
  Scatter plots ... 14
  Table ... 15
Sensitivity analysis ... 16
  Figure: T/S ratio, no comorbid depression and anxiety disorder ... 16
  Table: T/S ratio, no comorbid depression and anxiety disorder ... 17
Sensitivity analysis stratified by antidepressant medication use ... 18
  Figure: T/S ratio, no comorbid depression and anxiety disorder by antidepressant medication use 18
  Table: T/S ratio, no comorbid depression and anxiety disorder by antidepressant medication use . 19

GWAS summary statistics

Table S1. GWAS summary statistics

**Phenotype**	**GWAS reference**	**Sample size**	
		Cases	Controls
Anxiety disorder	Otowa et al. (2016) doi: 10.1038/mp.2015.197	7016	14745
Major depressive disorder	Wray et al. (2018) * doi: 10.1038/s41588-018-0090-3	45591	97674
Bipolar disorder	Stahl et al. (2019) doi: 10.1038/s41588-019-0397-8	20352	31358

Note: GWAS = genome wide association study. *Summary statistics from Wray et al. (2018) excluding UK Biobank and 23andMe data.

Covariates

Table S2. Covariates

UK Biobank data field	Variable
21003	Age at baseline assessment
31	Sex
30000	White blood cell count (cells/litre) measured in whole blood using an automated, clinically validated, Coulter LH 750 System. Calibration and quality control were performed according to the manufacturer's recommendations.
189	The Townsend deprivation index is a neighbourhood-level measure of deprivation derived from national census data on car ownership, household overcrowding, home ownership, owner occupation and unemployment aggregated at residential postcodes. Higher values on the index reflect greater relative deprivation.
864,<br>884,<br>904	Physical activity was assessed using the International Physical Activity Questionnaire (IPAQ) short form, including data on the number of days per week spent walking, engaging in moderate-intensity physical activity (e.g., "carrying light loads, cycling at normal pace") or engaging in vigorous-intensity physical activity (i.e., "activities that make you sweat or breathe hard such as fast cycling, aerobics, heavy lifting") for ≥ 10 min continuously.
20116	Smoking status was assessed using two questions summarising current and past smoking behaviour. Individuals who responded "Yes, on most or all days" or "Only occasionally" to current tobacco smoking were coded as "current". Individuals who responded "Smoked on most or all days" or "Smoked occasionally" to past tobacco smoking were coded as "former". Individuals who responded "No" to current tobacco smoking and "Just tried once or twice" or "I have never smoked" to past tobacco smoking were coded as "never".
23104	Body mass index was calculated as weight divided by height squared (kg/m^2). Weight measurements were obtained with a Tanita BC-418 MA body composition analyser. Standing height measurements were obtained using a Seca 202 height measure.
23099	Body fat percentage was estimated by electrical bio-impedance with a Tanita BC-418 MA body composition analyser.
30710	Serum C-reactive protein levels (mg/litre) were measured by immunoturbidimetric high-sensitivity analysis on a Beckman Coulter AU5800.

T/S ratio in individuals with mental disorders

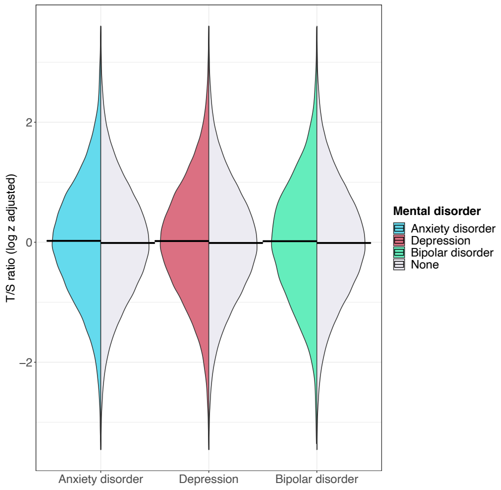

Figure S1. Average T/S ratio (log z adjusted) in individuals with and without mental disorders. Horizontal lines show group means. T/S ratio values below the 0.01st or above the 99.99th percentile not shown.

T/S ratio in individuals with any mental disorder by lithium use

Figure: T/S ratio by lithium use

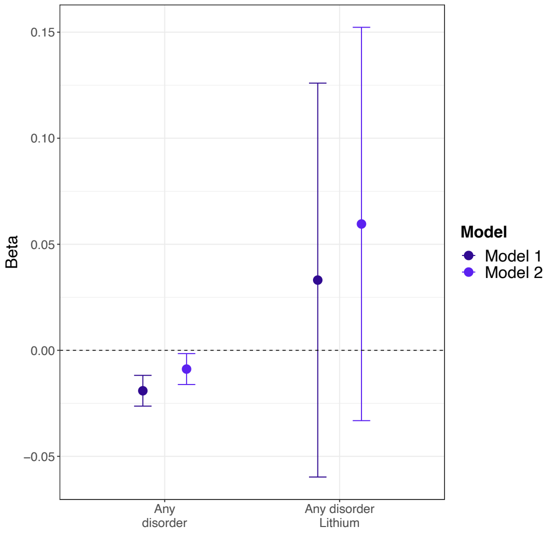

Figure S2. Average T/S ratio (log z adjusted) in individuals with any mental disorder (anxiety disorder, depression or bipolar disorder) compared to individuals without mental disorders (reference group) stratified by lithium use. Estimates shown are ordinary least squares regression beta coefficients and 95% confidence intervals. Model 1 – adjusted for age and sex; Model 2 – adjusted for age, sex, white blood cell count, Townsend deprivation index, physical activity, smoking status, body mass index, body fat percentage and C-reactive protein.

Table: T/S ratio by lithium use

Table S3. T/S ratio (log z adjusted) in individuals with any mental disorder by lithium use

	Model 1					Model 2				
Term	*β*	95% CI		$p_{Bonf.}$	p_{BH}	*β*	95% CI		$p_{Bonf.}$	p_{BH}
No disorder	Ref	-	-	-	-	Ref	-	-	-	-
Any disorder	-0.019	-0.026	-0.012	<0.001	<0.001	-0.009	-0.016	-0.002	0.070	0.035
+ Lithium	0.033	-0.060	0.126	>0.999	0.485	0.060	-0.033	0.152	0.832	0.277

Note: β = ordinary least squares regression beta coefficient; CI = confidence interval; Ref = reference group; Bonf. = Bonferroni; BH = Benjamini & Hochberg. Any disorder group included individuals with anxiety disorder, depression or bipolar disorder. Model 1 – adjusted for age and sex; Model 2 – adjusted for age, sex, white blood cell count, Townsend deprivation index, physical activity, smoking status, body mass index, body fat percentage and C-reactive protein. *P*-values corrected for four tests.

T/S ratio in individuals with mental disorders by antidepressant medication use

Figure: T/S ratio by antidepressant medication use

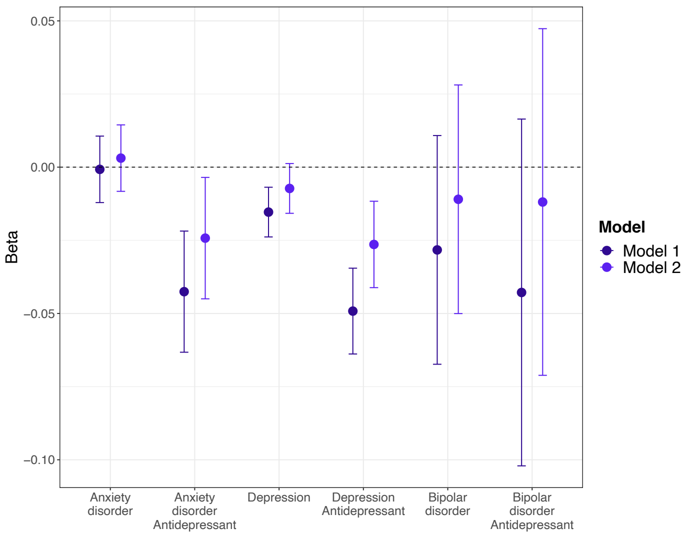

Figure S3. Average T/S ratio (log z adjusted) in individuals with mental disorders compared to individuals without mental disorders (reference group) stratified by antidepressant medication use. Estimates shown are ordinary least squares regression beta coefficients and 95% confidence intervals. Model 1 – adjusted for age and sex; Model 2 – adjusted for age, sex, white blood cell count, Townsend deprivation index, physical activity, smoking status, body mass index, body fat percentage and C-reactive protein.

Table: T/S ratio by antidepressant medication use

Table S4. T/S ratio (log z adjusted) in individuals with mental disorders by antidepressant medication use

	Model 1					Model 2				
Term	*β*	95% CI		$p_{Bonf.}$	p_{BH}	*β*	95% CI		$p_{Bonf.}$	p_{BH}
No disorder	Ref	-	-	-	-	Ref	-	-	-	-
Anxiety disorder	-0.001	-0.012	0.011	>0.999	0.898	0.003	-0.008	0.014	>0.999	0.712
+ Antidepressant	-0.043	-0.063	-0.022	<0.001	<0.001	-0.024	-0.045	-0.004	0.264	0.053
Depression	-0.015	-0.024	-0.007	0.005	0.001	-0.007	-0.016	0.001	>0.999	0.188
+ Antidepressant	-0.049	-0.064	-0.035	<0.001	<0.001	-0.026	-0.041	-0.012	0.005	0.001
Bipolar disorder	-0.028	-0.067	0.011	>0.999	0.235	-0.011	-0.05	0.028	>0.999	0.712
+ Antidepressant	-0.043	-0.102	0.016	>0.999	0.235	-0.012	-0.071	0.047	>0.999	0.757

Note: β = ordinary least squares regression beta coefficient; CI = confidence interval; Ref = reference group; Bonf. = Bonferroni; BH = Benjamini & Hochberg. Model 1 – adjusted for age and sex; Model 2 – adjusted for age, sex, white blood cell count, Townsend deprivation index, physical activity, smoking status, body mass index, body fat percentage and C-reactive protein. *P*-values corrected for 12 tests.

T/S ratio in individuals with mental disorders by antipsychotic medication use

Figure: T/S ratio by antipsychotic medication use

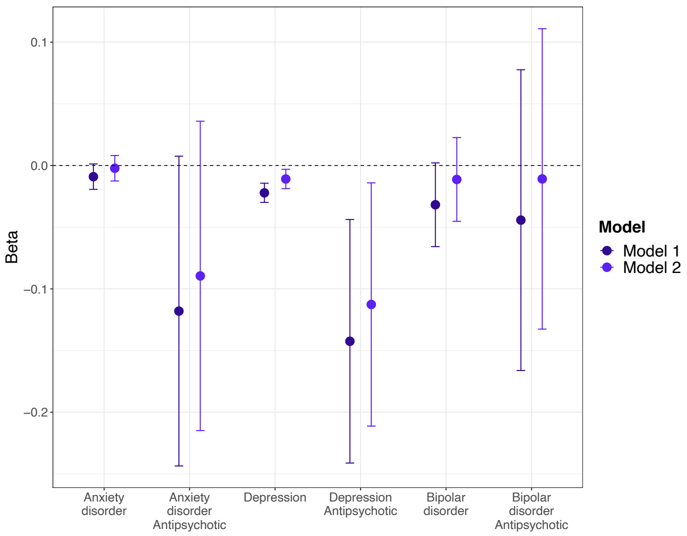

Figure S4. Average T/S ratio (log z adjusted) in individuals with mental disorders compared to individuals without mental disorders (reference group) stratified by antipsychotic medication use. Estimates shown are ordinary least squares regression beta coefficients and 95% confidence intervals. Model 1 – adjusted for age and sex; Model 2 – adjusted for age, sex, white blood cell count, Townsend deprivation index, physical activity, smoking status, body mass index, body fat percentage and C-reactive protein.

Table: T/S ratio by antipsychotic medication use

Table S5. T/S ratio (log z adjusted) in individuals with mental disorders by antipsychotic medication use

	Model 1					Model 2				
Term	*β*	95% CI		$p_{Bonf.}$	p_{BH}	*β*	95% CI		$p_{Bonf.}$	p_{BH}
No disorder	Ref	-	-	-	-	Ref	-	-	-	-
Anxiety disorder	-0.009	-0.019	0.001	>0.999	0.147	-0.002	-0.012	0.008	>0.999	0.738
+ Antipsychotic	-0.118	-0.244	0.008	0.786	0.133	-0.089	-0.215	0.036	>0.999	0.243
Depression	-0.022	-0.03	-0.014	<0.001	<0.001	-0.011	-0.019	-0.003	0.077	0.026
+ Antipsychotic	-0.142	-0.241	-0.044	0.056	0.026	-0.113	-0.211	-0.014	0.302	0.076
Bipolar disorder	-0.032	-0.066	0.002	0.798	0.133	-0.011	-0.045	0.023	>0.999	0.618
+ Antipsychotic	-0.044	-0.166	0.078	>0.999	0.618	-0.011	-0.133	0.111	>0.999	0.861

Note: β = ordinary least squares regression beta coefficient; CI = confidence interval; Ref = reference group; Bonf. = Bonferroni; BH = Benjamini & Hochberg. Model 1 – adjusted for age and sex; Model 2 – adjusted for age, sex, white blood cell count, Townsend deprivation index, physical activity, smoking status, body mass index, body fat percentage and C-reactive protein. *P*-values corrected for 12 tests.

Associations between age and T/S ratio in individuals with and without mental disorders

5-year age groups

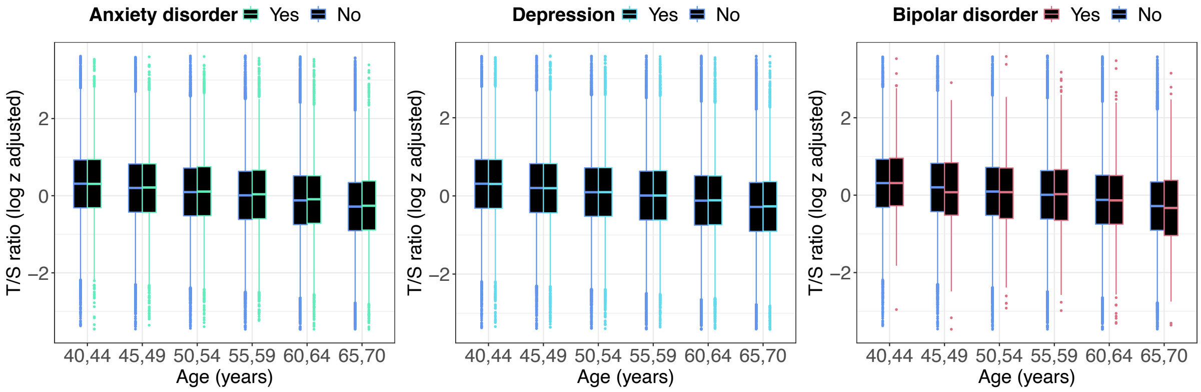

Figure S5. Age-related differences in average T/S ratio (log z adjusted) in individuals with and without mental disorders. T/S ratio values below the 0.01st or above the 99.99th percentile not shown.

Generalised additive models

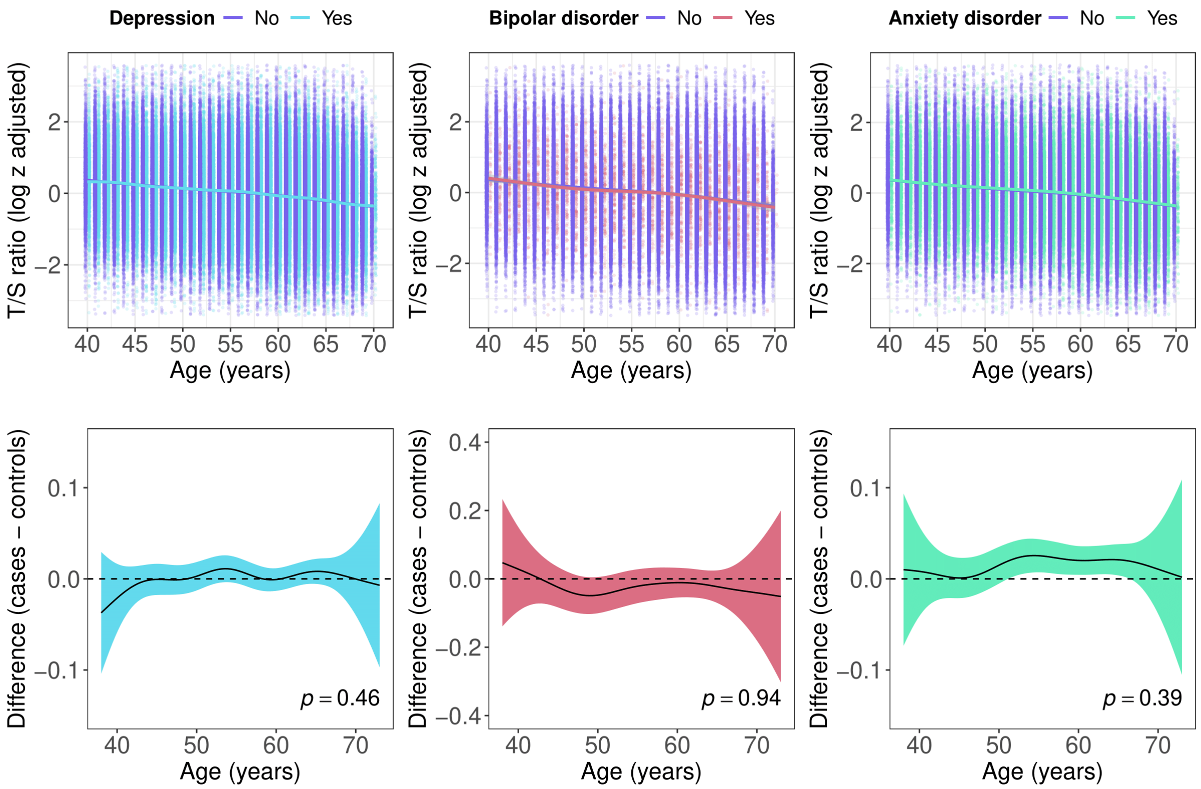

Figure S6. Top panels: scatter plots showing average T/S ratio (log z adjusted) by age in individuals with and without mental disorders. T/S ratio values below the 0.01st or above the 99.99th percentile not shown. Bottom panels: difference smooths comparing age-related differences in average T/S ratio (log z adjusted) of individuals with and without mental disorders. Positive values on the y-axes correspond to longer telomeres in individuals with mental disorders. The smooth curves were estimated using generalised additive models. The shaded areas correspond to 95% confidence intervals.

Polygenic risk scores in individuals with mental disorders

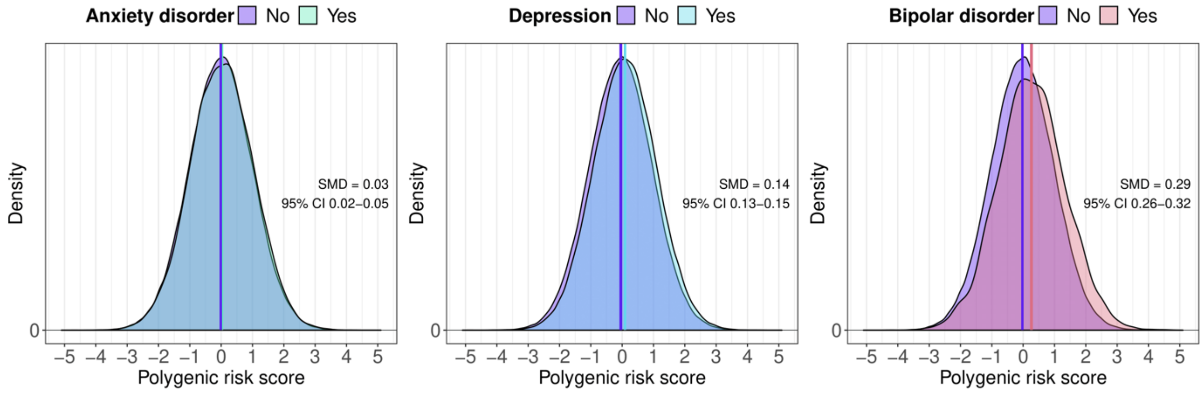

Figure S7. Differences in polygenic risk scores for anxiety disorder (left panel), depression (middle panel) and bipolar disorder (right panel) between individuals with these disorders and individuals without mental disorders.

Associations between T/S ratio and polygenic risk scores

Scatter plots

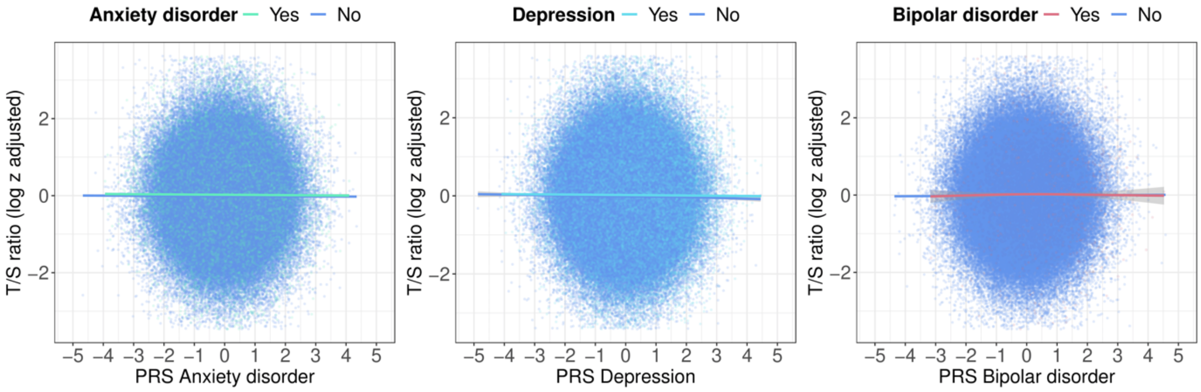

Figure S8. Associations between average T/S ratio (log z adjusted) and polygenic risk scores for anxiety disorder, depression and bipolar disorder. T/S ratio values below the 0.01st or above the 99.99th percentile not shown.

Table

Table S6. Associations between T/S ratio (log z adjusted) and polygenic risk scores for anxiety disorder, depression and bipolar disorder

	Full sample					Individuals with mental disorders					Individuals without mental disorders				
PRS	*β*	95% CI		$p_{Bonf.}$	p_{BH}	*β*	95% CI		$p_{Bonf.}$	p_{BH}	*β*	95% CI		$p_{Bonf.}$	p_{BH}
Anxiety disorder	-0.002	-0.006	0.001	0.589	0.196	-0.006	-0.015	0.004	>0.999	0.350	-0.002	-0.006	0.002	>0.999	0.385
Depression	-0.006	-0.010	-0.003	0.001	0.001	-0.006	-0.012	0.001	0.592	0.236	-0.008	-0.012	-0.004	0.001	0.001
Bipolar disorder	0.003	-0.001	0.008	0.342	0.171	-0.003	-0.036	0.030	>0.999	0.858	0.003	-0.001	0.008	0.707	0.236

Note: PRS = polygenic risk score; *β* = ordinary least squares regression beta coefficient; CI = confidence interval; Ref = reference group; Bonf. = Bonferroni; BH = Benjamini & Hochberg. All analyses were adjusted for the first six ancestry-informative population principal components, batch number and assessment centre. *P*-values adjusted for three (full sample) and six (individuals with and without mental disorders) tests.

Sensitivity analysis

Figure: T/S ratio, no comorbid depression and anxiety disorder

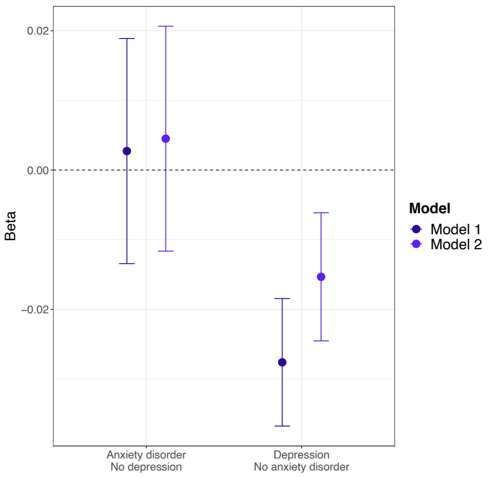

Figure S9. Average T/S ratio (log z adjusted) in individuals with anxiety disorder or depression compared to individuals without mental disorders (reference group), excluding individuals with comorbid anxiety disorder and depression. Estimates shown are ordinary least squares regression beta coefficients and 95% confidence intervals. Model 1 – adjusted for age and sex; Model 2 – adjusted for age, sex, white blood cell count, Townsend deprivation index, physical activity, smoking status, body mass index, body fat percentage and C-reactive protein.

Table: T/S ratio, no comorbid depression and anxiety disorder

Table S7. Average T/S ratio (log z adjusted), no comorbid depression and anxiety disorder

	Model 1					Model 2				
Term	β	95% CI		$p_{Bonf.}$	p_{BH}	β	95% CI		$p_{Bonf.}$	p_{BH}
No disorder	Ref	-	-	-	-	Ref	-	-	-	-
Anxiety disorder	0.003	-0.013	0.019	>0.999	0.741	0.004	-0.012	0.021	>0.999	0.741
Depression	-0.028	-0.037	-0.018	<0.001	<0.001	-0.015	-0.025	-0.006	0.004	0.002

Note: β = ordinary least squares regression beta coefficient; CI = confidence interval; Ref = reference group; Bonf. = Bonferroni; BH = Benjamini & Hochberg. Model 1 – adjusted for age and sex; Model 2 – adjusted for age, sex, white blood cell count, Townsend deprivation index, physical activity, smoking status, body mass index, body fat percentage and C-reactive protein. *P*-values corrected for four tests.

Sensitivity analysis stratified by antidepressant medication use

Figure: T/S ratio, no comorbid depression and anxiety disorder by antidepressant medication use

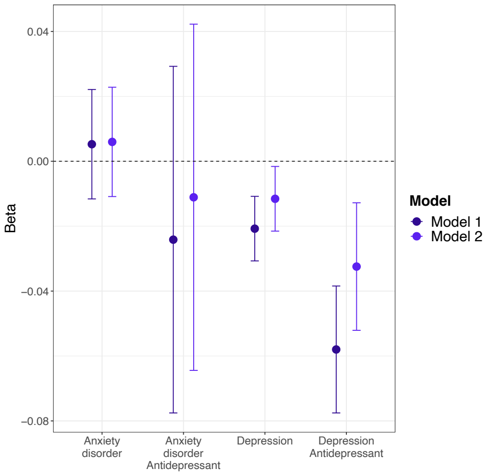

Figure S10. Average T/S ratio (log z adjusted) in individuals with anxiety disorder or depression compared to individuals without mental disorders (reference group) stratified by antidepressant medication use, excluding individuals with comorbid anxiety disorder and depression. Estimates shown are ordinary least squares regression beta coefficients and 95% confidence intervals. Model 1 – adjusted for age and sex; Model 2 – adjusted for age, sex, white blood cell count, Townsend deprivation index, physical activity, smoking status, body mass index, body fat percentage and C-reactive protein.

Table: T/S ratio, no comorbid depression and anxiety disorder by antidepressant medication use

Table S8. Average T/S ratio (log z adjusted), no comorbid depression and anxiety disorder, by antidepressant medication use

	Model 1					Model 2				
Term	β	95% CI		$p_{Bonf.}$	p_{BH}	β	95% CI		$p_{Bonf.}$	p_{BH}
No disorder	Ref	-	-	-	-	Ref	-	-	-	-
Anxiety disorder	0.005	-0.012	0.022	>0.999	0.618	0.006	-0.011	0.023	>0.999	0.618
+ Antidepressant	-0.024	-0.078	0.029	>0.999	0.601	-0.011	-0.064	0.042	>0.999	0.683
Depression	-0.021	-0.031	-0.011	<0.001	<0.001	-0.012	-0.022	-0.002	0.184	0.046
+ Antidepressant	-0.058	-0.078	-0.038	<0.001	<0.001	-0.032	-0.052	-0.013	0.010	0.003

Note: β = ordinary least squares regression beta coefficient; CI = confidence interval; Ref = reference group; Bonf. = Bonferroni; BH = Benjamini & Hochberg. Model 1 – adjusted for age and sex; Model 2 – adjusted for age, sex, white blood cell count, Townsend deprivation index, physical activity, smoking status, body mass index, body fat percentage and C-reactive protein. *P*-values corrected for eight tests.
